# Kounis and Takotsubo, Two Syndromes Bound by Adrenaline: The “ATAK” Complex

**DOI:** 10.1155/2023/7706104

**Published:** 2023-09-14

**Authors:** Ravi Vazirani Ballesteros, Juan Carlos Gómez Polo, Carmen Olmos, Isidre Vilacosta

**Affiliations:** Department of Cardiology, Cardiovascular Institute, Hospital Clínico San Carlos, Madrid, Spain

## Abstract

*Background*. The term “ATAK” complex has been coined by the association of adrenaline, takotsubo, anaphylaxis and Kounis syndrome. We present an uncommon case of an “ATAK” complex with biphasic onset and a midventricular takotsubo pattern. *Case Summary*. A 50-year-old male was brought to the emergency department in anaphylactic shock. He had progressive exertional dyspnea and angina for the past 2 days. The intravenous administration of adrenaline for anaphylactic shock resulted in chest pain and concerning ECG repolarization findings. The patient was immediately transferred to the catheterization laboratory. Coronary angiography showed a midventricular ballooning pattern without significant coronary stenosis, with subsequent recovery during hospitalization, suggestive of takotsubo syndrome. The allergy tests remained inconclusive for the trigger. *Discussion*. Adrenaline-mediated stress is the link between these two entities, in which Kounis syndrome itself or anaphylactic shock treatment (adrenaline) are potential triggers for takotsubo syndrome.

## 1. Introduction

Kounis syndrome is an acute coronary syndrome caused by the release of inflammatory cytokines due to a mast cell activation. It was introduced by Kounis and Zavras in 1991 as “allergic angina” or “allergic myocardial infarction”. Its relationship with takotsubo syndrome was recently described in the ATAK complex, which is coined by the association of adrenaline, takotsubo, anaphylaxis and Kounis syndrome.

We present an uncommon case of an “ATAK” complex with biphasic onset and a midventricular takotsubo pattern.

## 2. Case Presentation

A 50-year-old male is brought to the emergency department by his wife in anaphylactic shock.

He had no previous known allergies, with a past medical history of depression treated with alprazolam for over 7 years. He works as an accountant. He did not report any unusual food or animal contact (he had a hamster for over 5 years). He had been vaccinated with the first SARS-CoV-2 dose (Pfizer©) one week before being admitted to the hospital.

The day before admission, he started experiencing exertional angina during his usual walk (over 500 meters), without registered fever until 14 : 00 of the same day, experiencing angina during the first 200 meters of the same walk in the afternoon. At night, he experienced throbbing pain on the left side of his chest at rest before going to bed.

The patient woke up with an intense palpebral edema as well as erythematous and pruritic plaques in his neck and head, with oppressive left-sided chest pain at rest.

On his way to the hospital, he suffered one episode of syncope while seated on the vehicle, without chest pain or palpitations, with full recovery 10 seconds after, without confusion or dizziness.

Upon his arrival, he was immediately admitted into the emergency department, with a blood pressure (BP) of 60/30 mmHg and 68% of oxygen saturation. The ECG displayed on the monitor showed sinus tachycardia at 110 beats per minute (bpm).

On physical examination, he was pale, diaphoretic, and had a respiratory rate of 30 respirations per minute (rpm), without any other abnormal physical findings.

Anaphylactic shock was quickly suspected, and the patient was administered 0.5 mg of IV adrenaline in a bolus, 200 mg of hydrocortisone, and 1500 cc of crystalloids. Seconds after the infusion, the patient started experiencing a burning sensation radiating from his right arm (injection point) to the left side of his chest, which ceased after 20 seconds.

**Figure 1:**
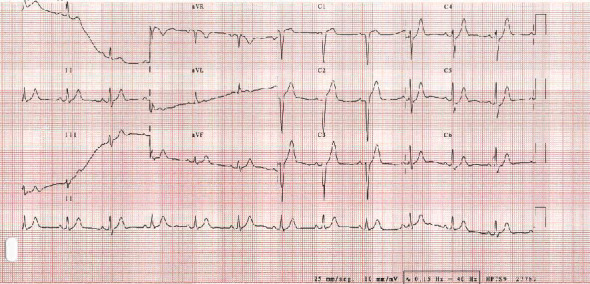
Initial ECG. Initial ECG showing a QS pattern in V1 and V2, as well as 1,5 mm of ST segment elevation in V1 and 3,5 mm in V2, with an ST segment depression in V4 (maximum of 1,5 mm), and V5 and V6 with a peaked T wave.

A 12-lead ECG is immediately performed (Figure 1), showing late R wave progression in the precordial leads as well as upsloping ST-segment depression and peaked T waves in the left precordial leads.

**Figure 2:**
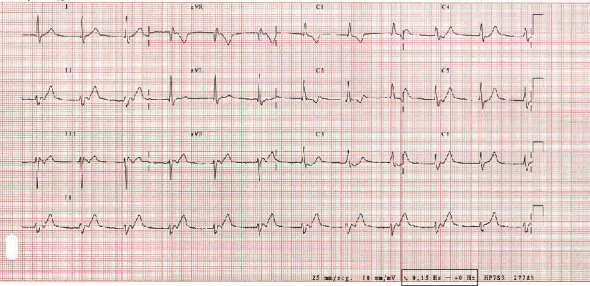
ECG performed 20 minutes after. ECG shows junctional rhythm at 70 bpm, with an incomplete right bundle branch block in V1 and disappearance of the ST segment elevation in V1 and V2;

An ECG was performed 20 minutes after (Figure 2) showing junctional rhythm alternating with normal sinus rhythm.

The echocardiogram showed akinesis of the middle segments and normal apical contractility, thus raising the suspicion for midventricular stress cardiomyopathy (takotsubo syndrome).

Blood analysis showed a triptase of 12 (normal range within 3,5-11,4) with 6900 leucocytes (normal range 3000-12000) without neutrophilia or eosinophilia, without hs-TnI elevation at first, and a peak troponin of 1067 ng/mL (normal range ≤56 ng/mL) in the second measurement taken 3 hours since arrival. Leukocytes were normal as well (9100, normal range 4000-12000) without eosinophilia.

**Figure 3:**
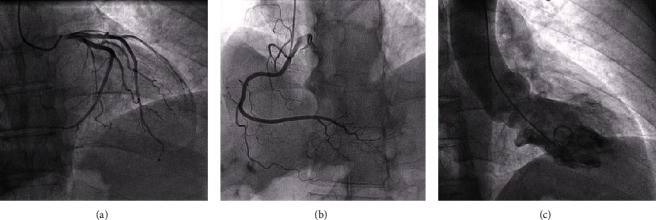
Coronary angiography. The angiography, performed after 2 h of admission in the ICU, shows (a–c) left coronary circulation without lesions, dominant right coronary artery without lesions, and diagnostic ventriculography with akinesis of the medium segments with apical preservation, suggestive of midventricular takotsubo cardiomyopathy.

The patient was transferred to the catheterization laboratory, and the angiography showed (Figure 3) no significant coronary artery stenosis and a midventricular takotsubo pattern in the ventriculography.

The echocardiogram was repeated two days after with normal contractility and a preserved systolic peak strain, thus making the diagnosis of takotsubo cardiomyopathy. He was started on oral prednisone.

Allergy team was consulted and advised to gradually lower the dose of prednisone, associate ebastine, and suspend alprazolam until further studies were performed.

The ECG was repeated prior to discharge with resolution of the repolarization abnormalities. The patient remained pain-free and was discharged.

His final diagnosis was the “ATAK” complex, formed by a type I Kounis syndrome of hatched onset and a takotsubo syndrome probably because of the intravenous supratherapeutic dose of adrenaline used to treat the anaphylactic shock.

## 3. Discussion

The ATAK complex is a truly diagnostic and therapeutic challenge [1] and is defined by the concurrence of adrenaline-mediated stress, takotsubo, anaphylaxis, and Kounis syndrome.

The adrenergic insult generated by the Kounis syndrome probably diminished the threshold for the next adrenergic stimulus—in our case, a supranormal dose of intravenous adrenaline—to be able to induce the stress cardiomyopathy. In anaphylactic shock, the European Resuscitation Council (ERC) Guidelines of 2021 recommend 0.5 mg of intramuscular adrenaline and, in refractory cases, an IV dose of 20-50 mcg [2].

Type I Kounis syndrome is the most frequent variant (72.6% of cases) and consists of a coronary artery spasm due to the liberation of inflammatory mediators with or without cardiac enzyme elevation [3, 4]. Type II Kounis syndrome is observed in patients with preexisting atheromatous disease in whom the release of these mediators would also produce a coronary vasospasm, which can result on the rupture of the atheromatous plaque, manifesting as an acute myocardial infarction [5]. Type III occurs in the presence of a coronary artery stent, where the release of inflammatory mediators results in stent thrombosis [6]. Given that the coronary artery spasm is a classically hyperacute disease. The progressive and hatched onset in our case raised a low suspicion index for type I Kounis syndrome. The ECG changes could manifest involvement of the left anterior descending artery, thus causing this transient ECG pattern.

Soufras and Kounis [7] state that the mastocyte could suffer IgE-mediated degranulation due to synergic effect of different causal agents, thus allowing different antigens to accelerate the appearance of a serious allergic reaction in this type of patients. In 50% of the cases (as it is our case), the antigen is not clearly identified. The heterogeneity of the causal agents is a known fact, with cases described in the literature due to gelafundin administration, pheocromocytoma, hypocortisolism [8], and among others. The common link to all of them seems to be adrenalin-mediated stress.

The “ATAK” complex raises clinical questions such as what is the middle- and long-term prognosis of these patients or if there are specific long-term therapies to avoid recurrences.

Kounis et al. [8] question the use of adrenalin in these patients, as all the available preparations use sodium metabisulfite as a preservative, which has been described to cause anaphylactic shock, thus raising concerns about adrenalin and its dosage in this type of patients.

The patient's tests in the outpatient allergy clinic were negative for alprazolam and for animals (i.e., hamsters) and neither total IgE nor specific Anisakis IgE were elevated. Nevertheless, the possibility of a reaction to the COVID-19 vaccine could not be excluded, and he received subsequent doses of the vaccine without further events. The patient has not suffered any recurrence up to date.

## Data Availability

Data is available on reasonable request.
